# Disrupting the “empathy machine”: The power and perils of virtual reality in addressing social issues

**DOI:** 10.3389/fpsyg.2022.814565

**Published:** 2022-09-26

**Authors:** Carles Sora-Domenjó

**Affiliations:** Image Processing and Multimedia Technology Center, Universitat Politècnica de Catalunya-Barcelona Tech, Terrassa, Spain

**Keywords:** virtual reality, empathy, storytelling, films, prosocial behaviour, documentary, media effects, embodiment

## Abstract

This article looks through a critical media lens at mediated effects and ethical concerns of virtual reality (VR) applications that explore personal and social issues through embodiment and storytelling. In recent years, the press, immersive media practitioners and researchers have promoted the potential of virtual reality storytelling to foster empathy. This research offers an interdisciplinary narrative review, with an evidence-based approach to challenge the assumptions that VR films elicit empathy in the participant—what I refer to as the VR-empathy model. A review of literature from the fields of psychology, computer science, embodiment, medicine, and virtual reality was carried out to question and counter these claims through case studies of both fiction and non-fiction VR experiences. The results reveal that there is little empirical evidence of a correlation between VR exposure and an increase in empathy that motivates pro-social behavior, and a lack of research covering VR films exposure eliciting empathy. Furthermore, the results show an alarming lack of research into the long-term effects of VR films and other VR immersive experiences. This contribution aims to understand and demystify the current “empathy machine” rhetoric and calls for more rigorous, scientific research that can authenticate future claims and systemize ethical best practices.

## Introduction

During the most recent wave of VR films creation, a problematic discourse has emerged in the tech media press, and also among new media tech entrepreneurs and researchers (Bloom, 2017; Bertrand et al., 2018; Sánchez Laws, 2020): the potential for VR and immersive films to enhance empathy within others, and thereby promote pro-social behaviors. Although numerous articles have been published to demystify this controversial model, particularly in the context of non-fiction VR (Sutherland, 2016; Bollmer, 2017; Nash, 2017; Rose, 2018), a lack of an empirical body and cross-disciplinary reviews is detected. This work contributes to the discussion with and interdisciplinary and evidence-based review approach.

This narrative review aims to establish an interdisciplinary critical approach built upon empirical evidence and media theory taken from different research fields challenging the “empathy machine” rhetoric and its complex phenomena. By interrogating a scientific body of evidence and literature from different disciplines, a coherent set of concepts, categories and evidences are studied and confronted among them (Ragin, 1989), offering a multi-dimensional understanding of the object of study (Bogdan and Taylor, 1975) and contesting the model as a premature and unproved claim.

This article is divided into four parts. The first explains how the VR-empathy model was established. The second deconstructs empathy through the lens of psychology and neuroscience in order to obtain key aspects for exploring the VR empathy model. In the third section, I map the VR territory and its associated mediated effects, and present results from empirical studies that provide evidence that VR is a medium that could potentially affect subjects’ behavior and emotions eliciting empathy. I revisit seminal studies that employ VR to curtail implicit personal bias to reexamine the process by which they arrive at their conclusions. In the fourth and final section, I explore ethical concerns surrounding VR’s attempt to represent reality.

The conclusions of this research suggest that the empathy model must be scrutinized in designing VR storytelling experiences for complex social issues. At a time when VR is in the process of establishing itself as a mainstream medium, as Rose asserts, “the dominance of the empathy claim prove[s] a distraction, if not a barrier to development.” Therefore, efforts must be made to disrupt the false assumptions surrounding the VR empathy-model, which would result in more accurate empirical research and thus benefit researchers and creators working in VR films. It would also enrich content creation and production in the wider field.

## The rise of the “ultimate empathy machine”

Virtual worlds and metaverses, in their third wave of development, are again attracting the attention of the tech industry and consumers (Soler-Adillon and Sora, 2018). VR has a long history of development, beginning with its first technological attempts to create head-mounted displays (Sutherland, 1968) and immersive CAVE systems (Cruz-Neira et al., 1993) decades ago. During the last two waves (1968–2014) non-linear narratives were also developed, experimenting with new forms of digital storytelling (Laurel, 1993; Davenport and Murtaugh, 1995; Murray, 1997). What has changed is that consumers now have access to a variety of VR film editing tools, online video distribution platforms and VR headset technologies.

As mentioned, in the context of non-fiction virtual reality, there have been various attempts to create immersive experiences that increase our capacity for empathy to better enable participants to connect with one another. News magazines and industry literature have contributed to the hype through publications such as *Can VR Really Make You More Empathetic?* (Zhang, 2016) and *How to Create Empathy in VR* (Bailenson, 2018). Evangelists of VR maintain that this technology allows us to really get into another person’s shoes and feel how they live. They argue that VR, unlike previous media, through its technical capabilities, offers a more direct way to enter another person’s lived experience, bringing us closer to what cinema, theater, literature and other artistic media have attempted in the past.

During recent years the production of VR and immersive experiences that engage with the “real” has grown from a handful of more than 600 films listed in a *VR Non-fiction Mediography* (Bevan and Green, 2018). This, in turn, has significantly redefined the interest of VR films in festivals contents, media creation and press coverage.

The most covered topics of this genre appear to be diaspora, refugee camps and exile. In a quick YouTube search, one finds hundreds of videos using the term “refugee” with the filter “360° film.” These immsersive films often situate spectators in distant places in which participants can only shift their gaze and turn their head unable to affect the content. The first 360° film I found posted on YouTube is entitled “Burj Al Barajneh camp for Palestinian refugees—Lebanon. Aljazeera Media Training Center 360 Test” uploaded by Montaser Marai, a Palestinian-Jordanian journalist and documentary filmmaker who has worked for the news channel Al Jazeera since 2002. Viewers of 360 documentaries encounter personal and traumatic experiences, and attempt to foster an interpersonal relationship with the subjects they engage with—or inhabit. Deployed as part of broader communication campaigns for non-government organizations (NGOs), these documentarians seek to catalyze action and positive change in the world.

A central contradictory concept that has generated as many followers as detractors is the belief that VR is the “ultimate empathy machine,” a term popularized by Chris Milk in a very successful 2015 TED talk about VR. Milk, a renowned artist and currently the CEO of a VR company called *Within*, might have borrowed the concept from the film critic Roger Ebert who said about Hollywood: “[Cinema is] the most powerful empathy machine of all arts.” In his talk, Milk (2015) argued that VR “is a machine, but through this machine we become more compassionate, we become more empathetic, and we become more connected. And ultimately, we become more human.” Milk suggests that the technology itself, regardless of content, possesses an unparalleled capacity to create an empathetic connection with the protagonists in VR films.

Employing the “empathy-model” was a clever hook in the initial stages of VR, at least in terms of fundraising in economic forums, including Davos and the United Nations. And this might be due to the impact that the film *Clouds Over Sidra* (2015) had on the press that year. *Clouds Over Sidra* was the first VR documentary in the UN VR series. The film was made in collaboration with the company Here Be Dragons and was directed by Gabo Arora and Chris Milk, pioneers in this new wave of VR. It tells the story of the daily life of a 12-year-old Syrian refugee in the Za’atari camp in Jordan. The idea of VR as an empathy model entered the public discourse through world news outlets (*The New York Times, The Guardian*, PBS), NGOs and humanitarian organizations (United Nations and Doctors Without Borders) in their efforts to promote peacebuilding and social change. The United Nations even implemented a pilot program focused on broadening awareness of refugee resettlement and climate change, which was widely reported in the press and acclaimed at several festivals around the globe.

Initial discussions about the empathy machine often used the term empathy in a slippery, superficial way without differentiating empathy from sympathy and compassion. Nor was internal bias, or the general public’s understanding of empathy taken into consideration. As a result, critics of the model opposed the use of virtual reality to evoke empathy, especially when applied in the context of humanitarian crises. Some scholars say that empathy is strongly influenced and biased by factors such as race and similarity, and that in some cases it can backfire (Bloom, 2017), creating an aversion to groups, races or genders.

In this imprecise scenario—in terms of the complexity of the concept—supporters of VR still refer to it as the machine that will promote action because it connects the public so profoundly with its content. But how can we validate or confirm that all this is really happening? Even though the immersive feature of VR is mentioned as the main reason to elicit empathy, thus far scientific evidence shows mixed and inconclusive results (Tassinari et al., 2022) and there is not yet enough body of research to assert this model (Shin, 2018). Few empirical studies have assessed the empathetic responses created by the exposure to an immersive (VR or 360-degree video) storytelling experience in comparison with other non-immersive media (normal 2D video or images). And none of these studies found significant positive results concerning the capabilities of VR to foster long-term empathy (Archer and Finger, 2018; Farmer, 2019; Stelzmann et al., 2021). It remains then unclear whether immersion experiences foster an empathic appreciation of the other.

The lack of a solid understanding of what empathy means and the lack of empirical studies of VR experiences reveals the necessity to research the complexity of the concept. Avoiding the complexity of the phenomenon encourages the use of its more superficial and blurry meaning. The digital media industry might by applying—and even profiting from—this social framework when it refers to empathy and its vast potential in social development. However, its long-term goals for social engagement are not sufficiently clear.

## An introduction to empathy understanding

The notion of how we come to know others, of how we get close to others and understand their feelings and minds is the basis of empathy. Technology might have the potential of reshaping these processes by offering new interfaces that interfere in our communications, mediating and altering them. Singer, a neuroscientist who has published extensively about the neural basis of empathy, defines empathy as a *shared effect*, in the sense of feeling what another person is feeling (Singer and Lamm, 2009).

To understand the origins of empathy’s relationship with art, we can look back to the nineteenth century when the concept was coined by the psychologist Edward Titchener (1867–1927), as the translation of the German term “Einfühlung,” to describe the emotional “knowing” of a work of art; that is, an aesthetic experience of feeling an emotional resonance with a work of art. This can be defined as “a natural tendency to feel ourselves into what we perceive or imagine” (Riess, 2017).

Since then, empathy has been studied from different perspectives ranging from psychology and neurology to anthropology. The concept of empathy has produced significant differences of opinion and a lack of consensus regarding its nature across different disciplines, including psychology, ethnology and neuroscience (Sánchez Laws, 2020). There are almost as many definitions of empathy as scientists who have been studying the phenomenon for decades, and there are at least eight different major visions of neuroscientific theories of empathy (Batson, 2009). In fact, there is disagreement in the literature about its exact nature. Emotional, cognitive, and motivational views are involved, all to varying degrees (Preston and de Waal, 2002). There is even research on the dimensions of disagreement regarding empathy in the different perspectives presented in the literature (Pagotto, 2010). Debates are based on how empathy is produced, as a process or as an outcome, whether empathy is a cognitive state or an emotional state (Davis et al., 1994; Duan and Hill, 1996), whether we have the personal disposition to experience empathy, or whether it is a faculty that can be acquired.

What we currently know from psychology studies is that humans have a general empathetic response to the pain of others. Neurophysiological studies indicate that when people see or even imagine the pain of others, the brain activity is the same as if they were experiencing the observed pain themselves (Singer et al., 2006). And more recent research has even demonstrated with neuroimaging techniques that this feeling has real neuronal implications that activate attenuated limbic areas of the brain when we are aware of the pain of a loved one (Carr et al., 2003). Although it is true that several VR lab interventions reveal that some related aspects of empathy like implicit bias or perspective-taking are triggered (Peck et al., 2013; Banakou et al., 2016; Hofer et al., 2017; Ventura et al., 2020), so far there are no qualitative or quantitative indicators that might help investors, researchers or educators to agree that the VR storytelling medium is a solid model for boosting empathy with others, or at least not any more than older media, such as cinema or photography, that have extensively been discussed in media and communication studies (Gallese, 2015; Stadler, 2016).

It is also clear in the literature that VR responses are modulated by our interpersonal and social-cultural context and biases (Riess, 2017). Thus, these relevant emotions that help us to resonate with others are not a general behavior and involve inconsistency and unconscious bias. Due to its evolutionary presence in our species, empathy works better in groups that look similar to us: “Individuals tend to have the most empathy for others who look or act like them, for others who have suffered in a similar way, or for those who share a common goal.” [.] “The truth of the matter is that empathy is not always an equal opportunity benefactor” (Riess, 2017).

In our account of empathy, it is critical to consider the differences and nuances found in the literature. Singer points out an important difference between sympathy and empathy. For Singer and Lamm (2009), sympathy is the vicarious emotional reaction of feeling pity or sorrow *for* someone else’s misfortune, whereas empathy implies “feeling *with* the others,” sharing the same affective states. And when these shared emotions are felt, an “emotional contagion” effect can drive our emotion unconsciously. In a situation where we are sharing concerns for another person’s welfare, while concerned with their feelings, and thus being sympathetic, we cannot be experiencing the same emotional states as they are. These ideas of sympathy were also labeled in the literature as “empathetic concern” (de Waal, 2008).

As Hamilton-Giachritsis et al. (2018) argue, it is also important in the context of digital media to distinguish between the affective and cognitive aspects of empathy with the ability to see things from another person’s point of view—POV. This is considered a more cognitive and rational process and not necessarily related to emotions. This ability to project and imagine how another person is feeling has been widely studied in psychology as “perspective-taking” and offers highly useful results on empathizing with others. In this regard, as we will see in the next section, VR is an extremely effective platform for applying perspective-taking research, as it makes it possible to simulate different perspective-taking configurations.

In summary, scientific evidence shows us that empathy is a complex phenomenon that has different forms of understanding, where socio-cultural specifics must be taken into consideration. While there is evidence that our brain and body react to the pain and emotions of others, empirical evidence on how a medium like VR might modulate empathy is not well established in the scientific literature, and especially concerning VR cinematic experiences for which there is not yet a corpus of studies.

## Empathy and mediated effects in virtual reality environments

Research has shown in different kinds of VR interventions during the last two decades that immersive experiences can increase components related to empathy in particularly conditions. However, meta-analysis studies revealed that “VR was no more effective at increasing empathy than less technologically advanced empathy interventions such as reading about others and imagining their experiences” (Martingano et al., 2021). It has been demonstrated in VR research that in particular and experimental conditions alterations to one’s digital self-representation can have a significant impact on how a person behaves in a virtual environment and that affects also its behaviors and attitudes fostering some of the qualities of empathy (Yee and Bailenson, 2007; Fox et al., 2013; Peck et al., 2013; Banakou et al., 2016; Kruzan and Won, 2019). Similarly, virtual perspective-taking through VR has been shown to be an effective method for changing behaviors and reducing negative social stereotyping and implicit biases (Yee and Bailenson, 2006, 2007; Hofer et al., 2017; Van Loon et al., 2018; Ventura et al., 2020).

The role of perspective-taking in a VR experience has been used to reduce the bias of offenders toward the recognition of fearful faces, eliciting empathy to the victims. In a recent studies (Seinfeld et al., 2018, 2021) a group of offenders embodied a virtual female body in a narrated short scene of domestic violence, showing positive results on how the changing of perspective of aggressive population using immersive virtual reality can modify emotional recognition. Other seminal research (Peck et al., 2013; Banakou et al., 2020) showed how been embodied in a dark-skinned virtual body lead to a reduction of implicit bias, increasing consequently the empathy to the out-group. Along the lines of this research another study (Banakou et al., 2018) revealed that virtual reality can be used to enhance executive functioning while reducing also implicit bias indirectly. Authors design a study where young population embodied a virtual body of Einstein. Results showed that the embodiment of young adults in the older Einstein body led to a reduction of implicit bias against elderly population.

In this following section, I present a broad and balanced overview of the main VR effects related to empathy founded in literature: from implicit cultural and group bias, embodiment to perspective taking and virtual bodies. Although there exists evidence in how VR can mediate some qualities of empathy, as shown, cultural and personal contexts and particular VR features mediate partially our VR responses. It is relevant then to understand how personal factors are involved while studding mediated VR empathy effects in order to avoid generalizing the “VR Empathy machine model.”

Following this same approach, it is important also to remark that although all these studies tackle different perspectives of empathy and might be considered partially positive for the interrogation of the “Empathy machine model” a lack of storytelling approach design on all these VR experiments brings to light a preliminary conclusion on the need of more focused research of its storytelling components. It was decided to add in this section a few artistic research projects that embrace in its design part of the interrogations that we are discussing here as a way to complement empirical research still not validated.

### Cultural biases and empathetic stress

From the literature one can understand that cultural and personal implications affect VR experiences. It has been demonstrated in the literature that racial bias and stereotypes can alter our empathic reactions (Avenanti et al., 2010). Empathy in VR experiences using avatars can decrease if the avatar represents someone of another race or ethnicity than the user, e.g., “white” people are less likely to help black avatars (Maister et al., 2015). Even gender causes different correlations. Research that compared men and women’s emotional understanding of empathy has shown significant results in which women do substantially better than men (Schieman and Van Gundy, 2000).

Experimental research (Arceneaux, 2017) has demonstrated that in situations of stress and anxiety the empathy gap between the in-group and out-group increases, and therefore the possibilities of being empathic with out-groups decreases. Consequently, VR simulations in which audiences are momentarily experiencing extreme situations as if they were another person, might cause the opposite effect on the user’s emotions to the one the creators are trying to elicit. Disability simulations could also create negative effects, promoting indirect discrimination against the simulated group rather than empathy. As Silverman (2015) points out, short experiences of a simulation of disabled people can create, among other things, the false sensation that the entire life of these people is as deeply frustrated as it seems in the simulations, as they do not have the experience and the skills that disabled people acquire and develop while dealing with their disabilities. As Silverman notes, what is highlighted is “the initial drama of becoming blind rather than the realities of being blind.” The audience can even project their feelings onto the disabled individual’s life, as happened in a blindfold experiment: “The students also projected their negative experience onto blind people. Compared with control students, blindfolded students estimated that blind people experience more fear, anger, confusion, and distress on a daily basis” (Silverman, 2015).

Empathetic situations might produce negative effects and stress reactions during an empathetic resonance. “Empathetic stress” has been defined in science as an empathetic situation in which negative effects are triggered while sharing emotional resonance with others (Hoffman, 1987). When empathetic distress is strong, it can lead to personal distress, creating anxiety, fatigue and other adverse feelings that act as a barrier to empathetic attitudes toward others. The focus of attention is shifted from the other to the self. It can even create a backlash effect in which the user may blame the other for creating such empathic feelings.

VR research that focuses on racial discrimination can also uncover some insights into body avatar and out-group empathy. Some experiments (Peck et al., 2013; Maister et al., 2015) have demonstrated that it is possible to affect existing racial and social prejudices of out-groups by experimenting with digital body swaps using virtual reality. Furthermore, as the author states “shared body representations are thought to form the fundamental basis of empathy and our understanding of others’ emotions and actions” (Maister et al., 2015). Mel Slater’s group carried out research using body swap experiments for reducing implicit racial bias. In an experiment (Banakou et al., 2016), “white” people went into a virtual class embodying a “black” virtual body where a teacher was showing various *tai chi* movements. The results showed a perceptual body ownership over the virtual body that entailed a reduction in their implicit, unconscious racial bias of “black” people, in a short-term assessment.

As shown, personal, cultural and genre groups are normally an unconscious quality that biases our perception of others. When people become aware of this, it can increase or decrease mediated effects. This suggests that empathy responses from a VR experience might be modulated and differ depending on each person and their individual awareness of the emotional implications.

### The psychology of embodiment in virtual reality

VR has a long history in psychology and therapeutic interventions. VR systems have proven to be useful clinically for treating a variety of phobias and psychological disorders (Riva et al., 2019). Some of these include social phobias (Klinger et al., 2005), panic and anxiety disorders (such as acrophobia, fear of flying/driving, etc.) (Vincelli et al., 2003; Botella et al., 2004), as well as obsessive-compulsive or post-traumatic stress disorders (Gregg and Tarrier, 2007; Fox et al., 2009; Slater, 2009; Meyerbröker and Emmelkamp, 2010), and other addictive behaviors (Lee, 2004).

VR systems offer a highly flexible method for creating and manipulating scenarios that might be dangerous to simulate in real life (Bailenson et al., 2004). In psychological treatments it is possible to recreate a particular experience with modulated potential danger, harm or embarrassment for participants. Furthermore, VR provides the opportunity to physically place the viewer in a scenario through their virtual self-representation or embodiment in a different human or even non-human form.

Virtual reality’s effectiveness is recurrently defined in the literature as its ability to create the illusion of *presence* in the user. *Presence* can be defined as the state in which the virtual world and its elements are experienced as if they were real (Lee, 2004; Slater, 2009). The illusion of *presence* has been characterized by two components: (a) *Place illusion*, which is defined as a powerful illusion of being in a place, despite knowing you are not there; and (b) *Plausibility illusion*, to the extent that what is happening seems like it is really happening (Slater, 2009). Furthermore, research has suggested that under the right multisensory conditions (such as 1PP, synchronous visuotactile and/or visuomotor contingencies), VR has the ability to generate in participants the illusion of *body ownership*, where the participant experiences an artificial body as if it were their own (even though it may look nothing like their real body).

### Perspective-taking in virtual reality

Extensive previous research in psychology has shown that assuming the perspective of someone else is an effective method for promoting empathy and pro-social behaviors (Underwood and Moore, 1982). Taking on the perspective of an individual of an out-group and imagining what it would be like to live this person’s life can decrease stereotypical conceptions of the individual, and can even increase empathy for their entire group (Batson et al., 1987). According to Herrera et al. (2018), there are different effects (even neuronal responses) depending on the type of perspective-taking used. “Imagine-other” (imagining another’s situation) perspective-taking leads to empathy and an altruistic motivation to help. But “Imagine-self” (how a participant would feel in that situation) perspective-taking tasks can lead to empathy, but also to personal distress and even an egoistic motivation to help. The positive outcomes of using the perspective-taking approach can also backfire and lead to negative outcomes, increasing the stereotyping of others. Previous research has shown that when people are asked to take the perspective of out-groups or “competitors” they become less empathetic toward them (Pierce et al., 2013), confirming the scientific consensus that the “positive effects of perspective-taking” are contextually bound (Herrera et al., 2018).

Considering that the qualities of perspective-taking (in particular contexts) might be beneficial for decreasing intergroup conflicts and increasing empathy, it is still not clear whether a VR mediated perspective-taking experience in films can lead to the same results. Before VR became an interesting medium for mediating empathy, previous experiments were carried out with other media, like video games (serious games). Role-playing has been used—without significant results—for empathy training in the context of interactive media and digital games (Gutierrez et al., 2014). What is new about the perspective-taking research applied in VR is the main core components of digital media experiences: interactivity and immersion. Whereas there is evidence that the qualities of digital media provide a more engaging experience by eliciting the sensation of *presence* and with it, perhaps, the feeling of taking the perspective of others, little research has been carried out into how perspective-taking operates in video games, interactive media or VR (Darvasi, 2016; Hasson et al., 2019), especially concerning the long-term effects.

In a recent study (Herrera et al., 2018), one of the most longitudinal and broadest sample in a VR study examining empathy (more than 500 participants ranging from 15 to 88 years old) compared the short- and long-term (8 weeks) effects of a traditional narrative perspective-taking task and a VR perspective-taking task. Participants were shown the VR film “Becoming Homeless,” a 7-min VR experience developed by the Virtual Human Interaction Lab at Stanford University. Control non-immersive experience conditions involved reading a text or interacting with a two-dimensional version of the experience. As expected, all participants under all conditions increased their level of empathy toward the homeless, but the VR condition had more positive and lasting results. However, the authors state that in the course of the 8 weeks after the exposure, the difference between the conditions (VR and non-VR) dissipated in self-reported empathy for the homeless, while positive attitudes toward the group increased significantly over time in the VR condition.

Other recent research also tested the capacity of using different perspective-taking (point of view) configurations in one immersive 360° video experience for effectively promoting pro-social emotions and behavior toward out-groups (Hasson et al., 2019). In a VR scene, participants see a 1-min Israeli-Palestinian confrontation between an Israeli soldier and a Palestinian couple at a military checkpoint from different points of view. The results show that immersive exposure to the rival out-group’s POV leads to more positive empathy and attitudes toward the out-group (Palestinians) than participants in the control condition (intragroup immersion). What is even more significant about this study are the long-term measures taken 5 months later, which revealed lasting real-life effects. In contrast to this study, more recent research (Hasler et al., 2021) that studied if immersive 360° videos elicits higher sense of presence and engagement, affecting ingroup’s moral judgment actions, concluded that the role of perspective-taking that can lead to empathy in the immersive experience do not differ significantly compared to watching a 2D video of the same scenario. Nevertheless, this study showed that an immersive experience can create greater impact and emotional arousal than traditional media.

These last studies are two of the first experiments that depicted long-term empathetic responses in a VR lab experiment, using different POV configurations in comparison with other 2D media. They therefore offer an optimistic perspective for our discussion and emphasizes the need to more research to understand the real potential of VR immersive experiences in the context of social change communication.

### Virtual bodies and representations in non-fiction films

Research has shown that the type of virtual body influences attitudes and behaviors (Slater and Banakou, 2021). In VR environments viewers can create a strong ownership illusion over virtual bodies (Ehrsson, 2007), and with it, get closer to another person’s emotions or situations by sharing with them the same space (de la Peña et al., 2010; Shin, 2018). Fully immersive VR experiences can offer users a sense of embodiment, through which they see themselves as part of the VR environment and feel that the VR avatar is a part of their own bodies (Trentini, 2015). However, most VR documentaries do not offer any kind of self-avatar representations during the experience. As recent research has shown in a study conducted with 150 titles of non-fiction VR films (Bevan et al., 2019), only 10 provided visible body representations. These results might eventually change as time goes on. However, as has been demonstrated since the 90s, it is very important to have a representation of your body during a virtual reality experience for creating the sensation of *presence*. As Slater (2009) says, “the action involved in looking at your own body provides very powerful evidence for PI (*place illusion*).”

In other early experiments the “Proteus effect” has been observed where the shape of the avatar representation can affect the way the participant behaves (Yee and Bailenson, 2007). In their study they demonstrated that the characteristics of self-representations, the digital avatars, have an influence on the way the users interact with other users, using their preconceptions of their assigned avatars’ representation and transferring their behaviors onto the physical features of the avatar. For instance, when the avatar is tall, participants behaved more confidently, and with a more attractive avatar they behaved in a more social way. It is necessary then to understand that the way the user is represented in the experience affects the user’s behavior, ultimately mediating the emotional effects on the participant.

One example of a non-fiction VR experience with body avatars is the VR documentary *The Enemy* by Ben Khelifa (2016). *The Enemy* is a room-scale virtual reality installation made in collaboration with the Open Documentary Lab at the Massachusetts Institute of Technology. In this installation, participants can observe a face-to-face encounter between two combatants in three different conflict zones: in El Salvador with the “maras,” in the Democratic Republic of the Congo, and in Israel and Palestine. In the experience, the audience can move physically and freely with a headset into three different spaces where the two combatants in each conflict express their feelings and their concerns about the conflict. While users do not have a self-representation of their own bodies, other participants are represented with abstract avatars to avoid collisions. In this project, the feeling of knowing that in the eyes of others you are being represented with an avatar is reinforced by the agency of the experience created by the system acknowledging the presence of the user. When you, the participant, are close enough to protagonists, they start talking to you and directing their gaze toward your position.

Another example of a project that uses previous evidence in virtual representations to challenge empathetic responses in a VR experience is the Hyphen-Labs artistic project *Neuro Speculative Afrofeminism* (NSA). Hyphen-labs, an artistic collective [London-Turkey-NYC], has created a VR project that challenges racial out-group perception. In this work participants are transported to a futuristic beauty salon where all women, including the virtual avatar of the participant, are black, thus generating an immersive perception of our own body as black and creating a virtual space that denounces the unrepresented black female group in a digital landscape. Most VR “empathy machine” model films seek to expose a social reality by using immersive technologies that capture real life events. What NSA illustrates is that VR can also be used to propose fictional environments and avatars for addressing social issues, and that new paradigms of body representation and cognitive effects can arise. The distant imaginaries of sci-fi and fictional realities might represent an interesting alternative way for depicting social issues in VR.

Older than NSA, *The Machine to be Another* (TMBA) from *Be Another Lab*, a Barcelona-based art collective, is also relevant for this discussion. TMBA is an immersive body swap experiment in which the audience can “experience” the body of another person with the goal of promoting and training empathy (Bertrand et al., 2018). In this experience participants sit in pairs in two chairs with a VR headset that streams real-time images from cameras in their headsets. During the experience users can see, through the headset, the body of the other person in the room as if it were their own body. Crucial to this project are the two performers of the collective who guide you during the process of discovering your “alter” body by moving and touching your extremities at the same time as the other person in the room does.

As Sutherland has commented elsewhere, TMBA causes “proprioceptive transference” reactions, suggesting that this builds essentially embodied empathic responses rather than creating a more cognitive and imaginative empathic experience of the other. And this is precisely what Sutherland questions about VR: “it cannot reproduce internal states, only the physical conditions that might influence that” (Sutherland, 2016). Although we could consider that TMBA fails in fostering social empathy engagement consistently, it is relevant for discussing other directions that immersive media can also modulate. Performance, participatory community engagement workshops and other digital co-creative practices (Uricchio et al., 2016; Cizek and Uricchio, 2019) can enrich the experience and rhetoric of the potential of VR moving from an individual “empathy machine” experience to a collective social one. Social and collective VR experiences might facilitate a better empathic resonance.

As shown, the use of virtual embodiment in VR experiences in social films is scarce, although has a great potential in creating the illusion of body ownership. This might be an important feature while considering VR experiences that are designed to foster changes in behaviors. Slater and Banakou (2021) has recently proposed a paradigm to foster prosocial attitudes and behaviors based on previous research using virtual embodiment. They propose a new tool (*Golden Rule Embodiment Paradigm*) based on a double model of VR exposure where participants are first involved in causing harm to another person for later reexperience the action being embodied as the victim. This two steeps design leads to enhanced helping behaviors toward the victims (Slater and Banakou, 2021).

Finally, in terms of interactivity, according to Martingano et al. (2021), in the context of pro-social VR experiences that want to elicit empathy might be relevant to design experiences in which users are asked to reflect on the lives of the subjects by asking things about their lives or giving them interactive options that play imagination and cognitive empathy in action. This same meta-study reveals that the level of immersion and interactivity it’s not particularly relevant to increase empathy. Thus, the level of realistic virtual body representations might not be the central strategy to foster cognitive empathy. Rather, they propose to design experiences that actively involve users in the creation of the virtual environment, requiring the engagement of their own imagination (Martingano et al., 2021).

## Ethics of virtual reality

Having shed light on the many complex angles implied in the discussion of the VR empathy model, I would like to complement this article with an approach to its ethical implications and concerns. As shown by science, VR experiences might lead to empathetic responses or even empathetic stress in the audience. These results make it imperative to consider the ethical implications involved while designing VR experiences that involve social issues. Previous discussions on ethical and moral perspectives of “VR for change” are scarce (Madary and Metzinger, 2016) but the field is recently evolving quickly (Nash, 2017; Emblematic and Frontline, 2018; Cotton, 2021).

While some issues in VR can be evaluated with standard ethical protocols, such as the content exhibited, VR introduces several new additional risks that still need to be addressed: media embodiment, VR capacity to generate mind illusions, interaction with other participants in a virtual space, etc. End users need to be sure that VR experiences published in digital markets have been ethically assessed because the general public trusts the technology that the industry puts out into society. Special attention must be paid to vulnerable groups such as children or people with mental disorders. There is some evidence that children are more vulnerable to confusing a VR exposure with a real-life experience (Kenwright, 2019).

Regulating VR ethically is a complex matter, as developers and manufacturers constantly use and change their designs and products (Kenwright, 2019). In this uncertain scenario, it is urgent to encourage experimental designs that use emerging technologies such as VR with consequent ethical thinking procedures, to ensure safety and avoid harmful experiences. Early identification of ethical issues in VR using anticipatory methodologies (Brey, 2012) will help societies to advance in moral dilemmas and, ultimately, avoid undesirable consequences.

As Roquet notes (Roquet, 2020), VR experiences might be more accessible and satisfactory when audiences share the same “universal subject position” as the developers. The expectations, perceptions and understanding of a VR experience might rely on the extent to which a user “comes to a VR experience possessing the same universal perceptual habits that developers originally designed for.” This alignment between the designer and the end user might be crucial in contextualizing the excitement of the empathy-model adopted during the early stage. This model was mainly discussed in contexts where designers and audiences were similar subjects, sharing the same expectations during festivals, conferences and exhibitions. The question here is what happens when this model is used by audiences that are distant from this “subject position,” who know less about VR experiences or digital design or even do not fit physically and bodily as the “subject” of the experience the developers designed. VR and 360 films are currently becoming very popular so a large percentage of the audiences might not fit with this universal “subject” targeted by the designers. This divergence can create uncomfortable experiences that can even harm people. In the context of social change strategies, we must point out that it is crucial to consider this confrontation, as some communities targeted by VR social films may be very unfamiliar with digital and immersive technologies.

The majority of the dominant VR model experiences place the audience in the daily-lived experiences of others without further context, as mere observers and voyeurs. This is labeled sometimes as a new form of “poverty techno-porn.” This way of occupying virtually the space of others while witnessing other people’s lives involves several tensions related to the distance that we as observers have from the testimonies and stories depicted. Most of these projects occur in real scenarios of conflict. Research shows that 73% of VR non-fiction films produced consisted entirely of live-action 360-degree video (Bevan et al., 2019), with a passive observant role. As Bloom (2018), states in films such as the 360-degree documentary *Clouds Over Sidra*, the experience of being a refugee is fundamentally represented within the immediate physical environs of the refugee camp. However, as Bloom states, the awfulness of a refugee experience is not about a particular space, or refugee camp, rather it has more to do with the entire experience of having been forced to abandon your own home for hopeless reasons and travel with your family and belongings during days and weeks, if not months, trying to find a place to settle and start a new life. Bloom explains that putting the participant in the middle of one particular scene without contextualizing the struggle and suffering of the people sharing their testimonies as a whole, will not succeed in creating impact and empathy. In terms of spatial transportation, in the simulation of being placed in another physical space, there is also great tension between the attention that authors want to earn from the audience and the inherent temptation of exploring the surroundings, turning away from the testimonies and ignoring them.

VR non-fiction films place audiences at an intimate personal distance from the portrayed testimonies. 85% of VR non-fiction films produced include engagement with other people in what is considered an “interpersonal zone,” to use Hall’s terminology of interpersonal spaces (Bevan et al., 2019). Most of this production grants audiences a passive observant role, and a very small number are presented from a first-person perspective; however, those that do, are not empowering, but rather victimizing (Bevan et al., 2019). In the majority of VR films, participants are placed at very close and improper physical distances, described by Nash (2017) as an improper moral distance between the characters and the viewers. As a result, in these experiences we are breaking all social and interpersonal distances that facilitate the capacity to engage in our social environments.

In the genre of documentary practices, the authors’ moral responsibility in showing the public particular personal and social struggles is not always clear, and empirical studies of the effect of content on audiences show the complexity of their responses, as they move from compassion to voyeurism, identifying with the testimonies or denying this connection (Nash, 2017). However, it is assumed in “media witness” theories that media have the capacity to create a feeling of live-ness and co-presence, of “being there,” that can be correlated with active responses (Peters, 2009).

In a VR simulation where we occupy the space of others, the proper distance that helps us to distinguish the self from others undermines our capacity to analyze and judge the situation, since the collapsing of that distance by the cognitive inputs of a system trying to immerse the audience in the experience is fundamental to the VR experience. The proper distance in a VR experience, Nash (2017) says, would be a combination of proximity and distance: enough proximity and presence to appreciate the other person’s situation, but also enough distance to make it possible to focus in on our own responses in order to assess how the situation is affecting us and how we can respond to it. VR experiences offer the possibility of moving from a variety of distinct perspectives or points-of-view (POV), from first person, fly-on-the-wall to omniscient. Each of these perspectives creates different sensations and can eventually create diverse distances from the testimonies. This quality is developed in 50% of the VR non-fiction productions that use at least two or three POVs (Bevan et al., 2019). If we apply this discussion to VR-generated films, we also have to consider how this distance can be affected by the user’s agency and interactivity.

In a project called *Hunger in Los Angeles*, an immersive film directed by de la Peña (one of the founders of the “Immersive Journalism”), portrays a simulation of the experience of watching a man going into a diabetic coma at the door of a food bank in Los Angeles. It is a virtual reality film that uses computer generated images to recreate the space and the people present at the moment of the event, mixed with real audio documents from the scene. In this work, the user participation is limited to witnessing the scene and moving freely in the space as a kind of forensic witness, acting as a mere spectator of the scene without participating or having any agency or being able to mitigate the pain of others. According to De la Peña these instances of VR “embodied rhetoric” (de la Peña, 2014) have a great potential to trigger empathy in an audience. However, through the lens of proper distance we see here an example of what an improper distance might represent, as the participant is a witness to a severe event without a particular narrative frame and context for their presence. The audience does not control any aspect of the unfolding of the events or have enough context during the experience to reflect on the moral, social and political implications of what they are seeing.

As presented here, VR computer generated experiences can include a wide variety of configurations, from interaction and agency features to embodied and representative characteristics. We believe that all these aspects must be considered as potential mediators of proper distance, modulating the audience between proximity and distance throughout the experience.

Another moral critique is that VR improper distance might create alienating experiences that negate the other person’s life in favor of the participant’s ascent (Bollmer, 2017). This author claims that in VR there is the risk of converting the characters’ stories into experiences, creating objectifications of other people’s lives, which can be simulated and assimilated as experiences. He argues that the current VR empathy discussion should substitute the aesthetic experience for a more empathetic transcendence, an ethical constructor of the emotions. In response to this, Bollmer presents the conceptual process of *radical compassion.* He defines this as an “ethical stance that refuses any attempt to experience, or even completely understand, the experience of another” (Bollmer, 2017). This implies not feeling what they feel and not negating their experience in favor of our own experience, instead being open to it based on the emotion it arouses. This idea avoids the empathy model that constitutes a paradigm for absorbing the experience of the other by producing affective responses within participants’ bodies instead of a relational response.

Finally, given the clinical evidence that experimental VR applications might provoke a lasting psychological impact on subjects, as mentioned above, I consider imperative to cover ethical questions surrounding the audience’s experience. Apart from inducing motion sickness and other risks involved in VR previously covered by ethics research recent studies (Madary and Metzinger, 2016) have pointed to new considerations such as duration, content of the experience, new screening procedures, false sense of agency, explicit consent in data privacy, and long-term immersion in future social VR environments that may cause “social hallucinations.” Avoiding any risk of harming the audience should therefore always be considered.

Although a VR lab experiment and a VR film experience have different configurations, it is appropriate to keep in mind all the above-mentioned ethical considerations to avoid psychological trauma or re-traumatizing subjects with personal past experiences. This brings us to our final point: taking care of the participants in the entirety of their experience. A VR experience care protocol needs to be designed for audiences as they might be emotionally affected by the characters or the stories of the VR film. The protocol should start with the informed consent that a VR film experience might generate lasting behavioral influences on subjects and should follow with a designed set of actions for addressing people’s responses and affective reactions during and after the experience. Any festival, center or institution that showcases VR films in public should seriously consider adopting a pre- and post-care model.

## Discussion

This work has sought to shed light on ways of demystifying current controversial discourse of the empathetic mediated effects of immersive films, the so-called “empathy machine” model. An integrative and interdisciplinary literature review has been presented covering this complex phenomenon in a balanced manner. State of the art on VR empathy research is presented covering main empathy effects that mediate and modulate virtual reality experiences. The main contribution of this article is that based on current research it’s premature at this early stage to consider VR as a medium that elicits empathy over other media such as cinema, television or photography. Empirical evidence supporting the claim that immersive storytelling experiences enhances empathy is limited. Thus the “Empathy machine model” should be approached in a more complex and rigorous way.

It is clear from research that in particular conditions alterations to one’s digital self-representation can have a significant impact on how a person behaves in a virtual environment and that affects also its behaviors and attitudes fostering some of the qualities of empathy. Results also indicate a lack of consensus on considering VR as a storytelling medium that elicits empathy by its qualities of immersion. Empathy it’s a complex phenomenon where cultural and personal implications might affect VR experiences, modulating and differing the empathy awareness depending on each person. VR mechanisms do not enhance the understanding of another person’s feelings and does not automatically lead to greater arousal of empathy.

As showed on this article some virtual reality experiences designed to elicit empathy might generate negative back-fired effects in relation to the outgroup depending on the subjects and the experimental design. It has been widely demonstrated that in virtual reality films, empathy involves, at the very least, social, cultural and physical bias that might interfere with empathetic responses, and also that different technical configurations might be implicated in these affective responses. The role of interactivity and agency in the arousal of empathy in VR experiences using current technical configurations seems that it’s not of particularly relevance (Martingano et al., 2021).

As showed, previous research validating immersive video as a storytelling medium that enhance empathy is scarce (Archer and Finger, 2018; Hasler et al., 2021). Also, while a few results show that VR film experiences can modulate emotions and empathy for a short period of time to a particular group of people, long-term effects of VR exposure are still unclear, as researchers indicate (Madary and Metzinger, 2016). Based on previous effects in mobile and web exposures, one can predict that VR immersive technologies might eventually have the same or even worse results that affect the same limbic areas involved in empathic resonance. Considering this lack of solid long-term exposure studies for VR films, and taking into account results showing VR’s capacity to affect people’s behaviors, I would like to make an appeal for more longitudinal studies and further research that examines how VR film and by extension VR videogames exposure affects our brains and their related emotional responses in the long-term.

This article exposes several facets that need to be considered while designing VR experiences “for social good.” A relevant remark was made on the need to consider the “subject design position” in relation to the personal stories unfolded (particularly in VR social films) and the potential audience. All these approaches can be adopted as a VR design framework for future works. In addition, in these VR development stages, more ethical reflection on future consequences is necessary at a time when it is difficult to predict the impact of virtual reality on society, and its foreseeable effects. When possible, a pre- and post-care model must be adopted when VR experiences are designed for public gatherings. I believe that understanding VR as a social and collective experience might facilitate a better empathic resonance. VR could be designed as part of a collective reflection of the unfolded story, accompanied and contextualized with information and care, creating a more harmonious space for embracing salient resonances and emotions.

The moral, ethical and mediated effects implied in an immersive media experience of being in proximity to (or occupying) other people’s lives is something that previous medium such as cinema could not provide in the same way. Therefore, we have to explore the overall effects, which are still unknown, of this medium more responsibly. VR is undoubtedly a great medium for sharing affective experiences. Given that this new media format is reaching millions of people and its mass adoption seems imminent in the near future, and that experiments are now also trying to enhance empathy in Mixed and Augmented Reality (Kroma and Lachman, 2018), it is imperative not to take any aspect of it for granted. The critique of this article on the basis of the science of empathy in psychology and VR studies does not invalidate the potential of this medium to spread important social and personal issues in the world and its potential to elicit empathy in particular lab conditions.

## Author contributions

The author confirms being the sole contributor of this work and has approved it for publication.
